# Supplementary material for: Morphological Changes of 3T3 Cells under Simulated Microgravity
Source: Cells. 2024 Feb 15;13(4):344. doi: 10.3390/cells13040344 (PMC10887114; doi:10.3390/cells13040344)

Supplementary data

1. FSC value

Supplementary Table S1. FSC value was analyzed by Accuri C6 Plus Flow Cytometer

	**FSC value**	
No.	**Control**	**SMG**
1	9913640	10599481
2	9422590	10308102
3	8755613	10204156
4	9715873	10481732
5	9559523	10413872

2. Nuclear intensity

Supplementary Table S2. Nuclear intensity value was analyzed by Cell Cyle App. of Cytell Microscope.

No.	Control	SMG
1	1.31	1.40
2	1.23	1.27
3	1.32	1.33
4	1.27	1.27
5	1.39	1.32
6	1.34	1.28
7	1.41	1.25
8	1.41	1.17
9	1.41	1.22
10	1.45	1.32
11	1.38	1.28
12	1.50	1.29
13	1.39	1.36
14	1.33	1.30
15	1.31	1.36
16	1.30	1.36
17	1.37	1.32
18	1.40	1.37
19	1.34	1.34
20	1.25	1.28
21	1.32	1.32
22	1.39	1.27
23	1.36	1.32
24	1.41	1.31

3. Nuclear shape

Supplementary Table S3. Nuclear shape value was analyzed by Cell Cyle App. of Cytell Microscope.

No.	Control	SMG
1	0.924	0.926
2	0.923	0.923
3	0.927	0.930
4	0.928	0.924
5	0.928	0.928
6	0.927	0.929
7	0.929	0.927
8	0.935	0.928
9	0.932	0.928
10	0.932	0.931
11	0.935	0.934
12	0.934	0.926
13	0.932	0.923
14	0.930	0.923
15	0.929	0.923
16	0.928	0.931
17	0.933	0.925
18	0.932	0.928
19	0.929	0.931
20	0.932	0.924
21	0.927	0.922
22	0.931	0.925
23	0.933	0.928
24	0.930	0.924

4. Nuclear area

Supplementary Table S4. Nuclear area was analyzed by Cell Cyle App. of Cytell Microscope.

No.	Control	SMG
1	211.41	214.50
2	221.56	218.71
3	210.17	214.38
4	222.97	227.58
5	197.87	212.53
6	199.87	212.92
7	199.91	219.16
8	195.04	224.95
9	195.13	212.37
10	195.05	207.09
11	206.56	214.66
12	198.82	217.60
13	202.68	215.58
14	210.03	214.55
15	210.85	213.69
16	201.72	207.64
17	204.52	220.27
18	194.36	209.90
19	212.81	205.90
20	218.90	218.09
21	209.74	218.53
22	190.96	215.36
23	205.63	215.77
24	206.24	227.17

5. Cell cycle progression analyzed by Cytell Microscope

Supplementary Table S5. Cell cycle progression analyzed by Cytell Microscope

No.	Cell percentage									
	Control group					SMG group				
	<2n (%)	G0/G1 (%)	S (%)	G2/M (%)	>4n (%)	<2n (%)	G0/G1 (%)	S (%)	G2/M (%)	>4n (%)
1	1.17	63.2	10.8	19.5	5.29	1.09	62.2	12	19.5	5.25
2	1.5	64	10.1	20	4.48	1.69	66.8	11.4	15.8	4.29
3	1.01	63	10.5	20.3	5.23	1.38	65.2	11.2	17.6	4.68
4	1.01	61.6	10.9	21.8	4.73	1.37	63.1	12.5	18	5.03
5	0.86	65.4	9.54	19.4	4.81	1.01	66.8	11.4	16.2	4.53
6	1.34	65	11.2	18.5	4.01	3.56	66.5	11.2	14.9	3.81
7	0.88	62.4	11.4	20	5.28	2.4	66.9	11.1	15.4	4.26
8	0.68	65.2	10.5	19.5	4.1	3.56	68.7	11.1	13.2	3.48
9	0.66	64.5	10.8	19.6	4.38	4.12	68.9	10.9	12.9	3.21
10	1.14	62.3	11.6	20.2	4.83	2.29	68	10.8	15.1	3.84
11	1.12	63.2	11.1	20	4.6	1.78	68.4	11	15.4	3.39
12	1.16	57.3	15.2	20.6	5.78	1.68	66.1	11.6	16.3	4.26
13	0.77	63.1	9.85	21.4	4.97	0.95	63.5	11.2	18.8	5.53
14	0.73	63.3	9.9	21.6	4.49	1.54	66.3	10.6	17	4.53
15	1.08	63.3	10.3	20.4	4.87	1.71	64.9	10.2	18.1	5.19
16	1.41	66.9	9.5	18.3	3.84	1.63	66.8	10.8	16.5	4.21
17	1.19	62.3	12	19.4	5.15	1.25	64.7	11.7	17.3	4.96
18	0.85	66.6	9.71	18.6	4.22	1.37	65.7	11	17.2	4.77
19	0.73	62.5	10.8	20.8	5.14	1.16	68.5	10.7	15.7	3.92
20	2.45	62.2	11.5	19.7	4.09	1.65	66.4	10.9	16.4	4.72
21	1.27	64.7	11	18.9	4.08	1.32	65.4	10.3	17.8	5.13
22	1.19	67	9.59	17.8	4.36	1.22	69	10.6	15.1	4.11
23	0.96	63.4	10.9	20.4	4.33	1.16	66	11.3	17.1	4.44
24	0.94	60.3	12.1	21	5.65	1.28	62.5	12.8	17.5	5.98

6. Cell density

Supplementary Table S6. Cell density was analyzed by Cell Cyle App. of Cytell Microscope.

No.	Cell number/well	
	**Control**	**SMG**
1	14492	12977
2	16421	14299
3	16509	15478
4	14810	14187
5	15015	13580
6	16225	11818
7	12708	14100
8	14388	13522
9	15823	14778
10	12545	12277
11	13267	11869
12	12863	14425
13	15398	16449
14	15688	15111
15	16432	15532
16	17556	15265
17	15467	14784
18	15251	13222
19	14228	13822
20	14031	14776
21	14618	13057
22	16687	15021
23	15756	13034
24	13052	13468

7. Western blot analysis

7.1. Actin

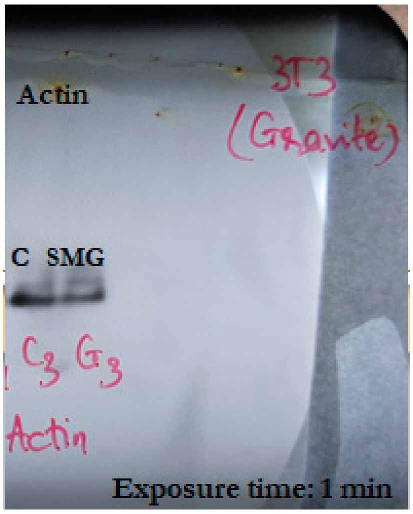

7.2. Tubulin

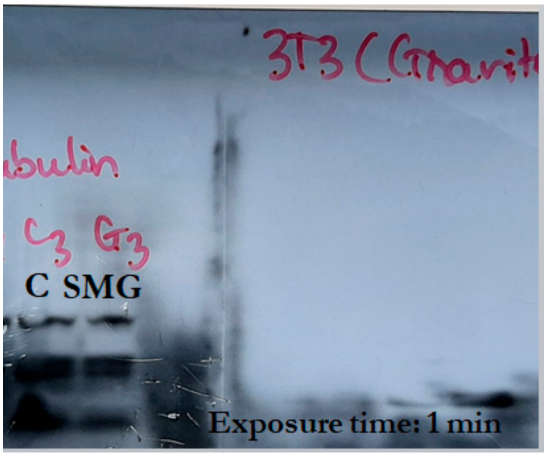

7.3. Cdk4

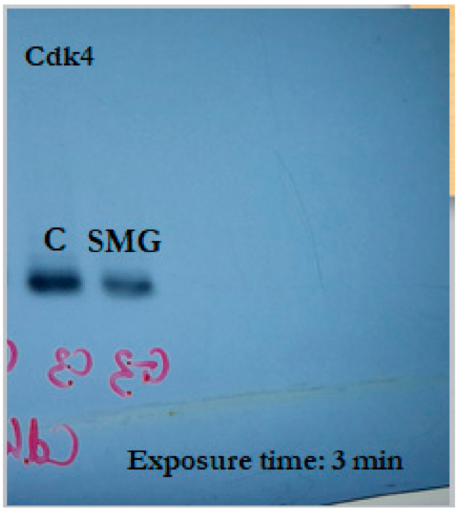

7.4. Cdk6

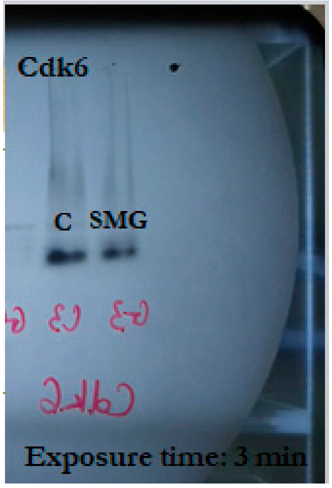

7.5. Gapdh

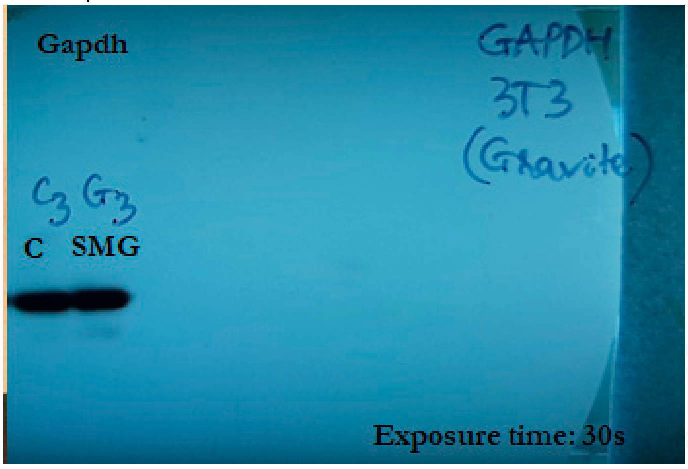

Supplement: Supplementary file 1 [file cells-13-00344-s001.zip › cells-2755706-supplementary.pdf]
